# Association Between Renal Fat Fraction and Early Biomarkers of Kidney Injury in Patients with Type 2 Diabetes Mellitus

**DOI:** 10.3390/jcm15083025

**Published:** 2026-04-15

**Authors:** Eisha Adnan, Lina Mao, Lingjun Sun, Yao Qin, Yangmei Zhou, Zhuo Chen, Tinghua Zan, Yun Mao, Tingting Luo, Shichun Huang, Xiangjun Chen, Zhihong Wang

**Affiliations:** 1 Department of Endocrinology, The First Affiliated Hospital of Chongqing Medical University, Chongqing 400016, China; mahereisha@gmail.com (E.A.); 15086643092@163.com (L.M.); sunlingjun_cu99@163.com (L.S.); qyclinical97@163.com (Y.Q.); q1219843728@163.com (Z.C.); zantinghua1124@163.com (T.Z.); luotingting268@163.com (T.L.); hsc931295238@163.com (S.H.); 2 Department of Clinical Nutrition, The First Affiliated Hospital of Chongqing Medical University, Chongqing 400016, China; 3 Department of Radiology, The First Affiliated Hospital of Chongqing Medical University, Chongqing 400016, China; maoyun1979@163.com

**Keywords:** renal fat fraction, chronic kidney disease, type 2 diabetes mellitus, kidney injury biomarkers magnetic resonance imaging

## Abstract

**Background:** Ectopic fat deposition has been demonstrated to play a critical role in the onset and progression of renal dysfunction. However, research on renal parenchymal fat deposition and its association with renal dysfunction in type 2 diabetes mellitus (T2DM) remains limited, particularly regarding its association with early kidney injury. The present study aimed to further investigate the relationship between renal fat fraction (FF) and biomarkers of kidney injury, thereby providing new evidence for the potential link between intrarenal fat accumulation and early renal impairment in T2DM. **Methods:** This cross-sectional study enrolled 60 patients with T2DM. Renal FF was quantitatively assessed using magnetic resonance imaging (MRI). Clinical characteristics, body composition parameters, and biochemical indices were collected. Levels of kidney injury biomarkers, including tumor necrosis factor receptors 1 (TNF-R1), tumor necrosis factor receptors 2 (TNF-R2), chitinase-3-like protein 1 (YKL-40), and kidney injury molecule-1 (KIM-1), were measured using enzyme-linked immunosorbent assay (ELISA). To evaluate the correlations between fat distribution and inflammatory biomarkers, Pearson correlation analysis was performed. Furthermore, linear regression analysis was conducted to explore the associations between renal FF and kidney injury biomarkers with adjustments for potential confounders such as smoking status, diabetes duration, and visceral fat. Lasso regression was used to screen variables. **Results:** The results demonstrated that renal FF was significantly positively correlated with serum YKL-40 (r = 0.3, *p* = 0.021), TNF-R1 (r = 0.246, *p* = 0.042), and urinary KIM-1 (r = 0.396, *p* = 0.004), indicating a close association between renal fat accumulation and early kidney injury biomarkers. In regression analyses adjusted for age, sex, and duration of diabetes, the associations between renal FF and these biomarkers remained significant. After further adjustment for potential confounders, including smoking history, alcohol consumption, hypertension, renin-angiotensin-aldosterone system (RAAS) inhibitors, sodium-dependent glucose transporters 2 (SGLT2) inhibitors, glucagon-Like Peptide-1 (GLP-1) receptor agonists, and lipid-lowering drugs, renal FF remained significantly associated with TNF-R1 (β = 0.327, *p* = 0.015), KIM-1 (β = 0.352, *p* = 0.021), and YKL-40 (β = 0.275, *p* = 0.025). Moreover, even after additional adjustment for visceral fat, the associations of renal FF with TNF-R1 and KIM-1 persisted. After using the Benjamini–Hochberg procedure for false discovery rate, the relationship between renal FF and KIM-1 had a significant difference. Variables of age and gender were excluded to build the parsimonious modeling using Lasso regression. It suggested that renal fat accumulation may contribute to kidney injury independently of visceral adiposity. **Conclusions:** The study systematically demonstrates a significant association between renal FF and early biomarkers of kidney injury in T2DM, which may suggest the potential role of renal fat accumulation in the pathogenesis of diabetic nephropathy. These findings provide clinical data support for the development of a fat-targeted intervention study. Future research should further elucidate the long-term mechanistic role of renal FF in diabetic nephropathy, as well as its potential value in early diagnosis and therapeutic applications.

## 1. Introduction

Chronic kidney disease (CKD) is a major global public health problem, and its incidence has risen significantly in recent years [1,2]. Type 2 diabetes mellitus (T2DM) is recognized as one of its most important etiological factors [3]. Accumulating evidence indicates that ectopic fat deposition plays a pivotal role in the onset and progression of renal dysfunction [4]. Among these, perirenal fat (PRF), due to its anatomical proximity to the kidneys, has attracted considerable attention and may contribute to renal injury through multiple mechanisms, including mechanical compression, lipotoxicity, and pro-inflammatory effects [5]. Clinical studies have shown that PRF accumulation is significantly associated with hypertension, proteinuria, and a decline in estimated glomerular filtration rate (eGFR) [6,7]. Beyond PRF, increasing attention has been directed toward the potential impact of intrarenal lipid deposition on renal function in recent years [8]. However, the association between renal fat fraction (FF) and early kidney injury remains insufficiently clarified. In addition, the underlying mechanisms by which renal lipotoxicity develops and progresses warrant further exploration. Novel biomarkers of renal injury include kidney injury molecule-1 (KIM-1), serum tumor necrosis factor receptors 1/2 (TNF-R1/TNF-R2), and chitinase-3-like protein 1 (YKL-40). KIM-1 primarily reflects early injury of the proximal renal tubules, whereas TNF-R1/TNF-R2 and YKL-40 are more indicative of systemic inflammatory responses and their relevance to renal disease progression [9,10,11]. These biomarkers have also demonstrated clinical value in studies of metabolic and obesity-related kidney disease [12]. In this study, renal FF was quantitatively assessed in patients with T2DM using magnetic resonance imaging (MRI), and its associations with early renal injury biomarkers were further analyzed. By adjusting for traditional metabolic and fat distribution factors, including smoking, duration of diabetes and hypertension, and visceral adiposity, the study aimed to clarify the relationship between renal FF and early kidney injury biomarkers. The findings are expected to enhance our understanding of the role of intrarenal lipid deposition in the pathogenesis and progression of diabetic kidney disease and to provide clinical data support for the development of fat-targeted therapeutic strategies.

## 2. Methods

### 2.1. Study Population

This study consecutively enrolled 60 patients with T2DM who were treated at the Department of Endocrinology, The First Affiliated Hospital of Chongqing Medical University, between June 2021 and May 2022 and who met the predefined inclusion criteria. The diagnosis of T2DM was based on 1999 World Health Organization criteria [13]. All participants provided written informed consent and underwent abdominal MRI. The exclusion criteria were as follows: (1) patients with a confirmed diagnosis of acute kidney injury; (2) patients with coexisting CKD, including renal artery stenosis, ischemic nephropathy, hypertensive nephropathy, chronic pyelonephritis, kidney stones with hydronephrosis, renal atrophy, or a solitary kidney; (3) patients with renal tumors, large renal cysts, or other conditions that could interfere with the measurement of renal FF; and (4) patients with a history of infection, immune system dysfunction, or malignancy. The study protocol was approved by the Ethics Committee of the First Affiliated Hospital of Chongqing Medical University (approval No. 2018-042).

### 2.2. Data Collection and Biochemical Assessment

Comprehensive medical histories were obtained from all participants, including smoking status, alcohol consumption, and major comorbidities. Anthropometry included height, weight, waist circumference (WC), systolic blood pressure (SBP), and diastolic blood pressure (DBP). Body mass index (BMI) was calculated as weight (kg) divided by height squared (m^2^). Body composition was assessed by trained technicians using dual-energy X-ray absorptiometry (DEXA; Hologic Discovery QDR series, Hologic Inc., Bedford, MA, USA), following standardized protocols. Participants were scanned in the supine position from head to toe in standard mode, with a scan duration of approximately 20 min and a scan width of 60 cm. Subcutaneous adipose tissue (SAT) and visceral adipose tissue (VAT) volumes were estimated using the Hologic whole-body DXA reference database and dedicated visceral fat analysis software.

For laboratory assessments, glycated hemoglobin (HbA1c) was measured by high-performance liquid chromatography. Fasting plasma glucose (FPG) and lipid profiles, including total cholesterol (TC), triglycerides (TG), high-density lipoprotein cholesterol (HDL-C), and low-density lipoprotein cholesterol (LDL-C), were determined using an automated biochemical analyzer (Model 7080, Hitachi, Tokyo, Japan) with enzymatic methods (reagents supplied by Leadman Biochemistry, Beijing, China). Serum creatinine, urinary creatinine, and urinary albumin were measured using a Roche Modular DDP automated biochemical analyzer (Roche Diagnostics, Mannheim, Germany), and the urinary albumin-to-creatinine ratio (UACR) was subsequently calculated.

### 2.3. Measurement of Renal Proton Density Fat Fraction

All MRI examinations in this study were performed using a 3.0T magnetic resonance imaging system (MAGNETOM Skyra, Siemens, Erlangen, Germany). Compared with ultrasound and CT, MRI precisely separates water signals from fat signals. The imaging protocol included a T1-weighted VIBE two-point Dixon sequence and a multi-echo Dixon VIBE sequence, both acquired during two consecutive breath-holds. The detailed parameters for the T1 VIBE two-point Dixon sequence were as follows: repetition time (TR) = 4.66 ms; echo times (TE) = 1.34 ms and 2.57 ms; slice thickness = 3 mm; total slices = 64; matrix size = 210 × 320; number of excitations = 1; field of view (FOV) = 400–450 mm × 87.5%; bandwidth = 820 Hz/pixel; total acquisition time = 17 s. For the multi-echo Dixon VIBE sequence, the parameters were TR = 9.46 ms; TE = 1.33, 2.64, 3.95, 5.26, 6.57, and 7.88 ms; slice thickness = 4 mm; total slices = 64; matrix size = 137 × 224; number of excitations = 1; FOV = 360–450 mm × 87.5%; bandwidth = 1060 Hz/pixel; acquisition time ≈ 20 s.

Following image acquisition, fat fraction (FF) maps and pure fat images were automatically generated using the post-processing toolkit in MRI system. Quantification of renal FF was performed with the syngo.via of VB20 version imaging software (Siemens Healthcare, Erlangen, Germany). For each kidney, the FF value was calculated as the arithmetic mean of five slices, and the final renal FF was obtained by averaging the values of both kidneys.

Image analysis was independently conducted by two radiologists (Reader A: L.H.X., 3 years of experience; Reader B: P.Y., 2 years of experience), both blinded to all clinical information. The measurement process was supervised by a senior abdominal radiologist (Y.M.) with extensive diagnostic experience. Inter-observer agreement was assessed using the intraclass correlation coefficient (ICC). ICC values were interpreted as follows: <0.5, poor agreement; 0.5–0.75, moderate agreement; 0.75–0.9, good agreement; and >0.9, excellent agreement [14]. In this study, the inter-observer reproducibility of renal FF measurement was good to excellent, with an ICC of 0.822.

### 2.4. Measurement of Kidney Injury Biomarkers

In this study, ELISA was employed to determine the expression levels of TNF-R1, TNF-R2, and KIM-1 in human serum (R&D system Inc., Minneapolis, MN, USA). All reagents and samples were equilibrated to room temperature prior to use.

For TNF-R1 detection, 200 µL of TNF-R1 conjugate was added to each well and incubated at room temperature for 2 h. After washing, 200 µL of substrate solution was added and incubated for 20 min in the dark, followed by the addition of 50 µL stop solution. Optical density was measured at 450 nm with correction at 570 nm.

For TNF-R2 detection, 50 µL of assay diluent was added to each well, followed by 200 µL of standards, controls, or samples. Plates were incubated for 2 h and washed three times, after which 200 µL of human TNF-R2 conjugate was added and incubated for another 2 h. Following repeated washing, substrate solution was added and incubated for 20 min in the dark, then stop solution was applied, and absorbance was measured.

For KIM-1 detection, 100 µL of assay diluent and 50 µL of sample were added to each well and incubated for 2 h. After four washes, 200 µL of human KIM-1 conjugate was added and incubated for 2 h. Plates were washed again, followed by the addition of substrate solution and incubation for 30 min in the dark. After adding stop solution, absorbance was recorded at 450 nm with correction at 570 nm.

All procedures were performed strictly in accordance with the manufacturer’s instructions, and measured samples twice. Averages were used as the biomarker data.

### 2.5. Statistical Analysis

All statistical analyses were performed using SPSS version 26.0 software (IBM Corp., Armonk, NY, USA). Renal FF was stratified into tertiles, and participants were accordingly categorized into three groups: ‘low,’ ‘medium,’ and ‘high’ levels. Continuous variables were expressed as mean ± standard deviation (mean ± SD) or median with interquartile range (Q1, Q3), and group comparisons were conducted using one-way analysis of variance (ANOVA) or the Kruskal–Wallis test, as appropriate. Categorical variables were presented as frequencies or percentages and compared using the Chi-square test.

Pearson correlation analysis was applied to assess the associations between fat distribution and inflammatory biomarkers. To further evaluate the relationship between renal FF and inflammatory markers, four linear regression models were constructed with progressive adjustment for potential confounding factors according to the study design. Lasso regression analysis was used to perform variable selection and model simplification. A two-tailed *p* value < 0.05 was considered statistically significant.

## 3. Results

### 3.1. Clinical Characteristics of Participants

A total of 60 patients were enrolled in this study. There were 26 patients with eGFR ≥ 90, 22 patients with eGFR between 60 and 90, 11 patients with eGFR between 30 and 60, and 1 patient with eGFR < 30. In addition, there were 31 patients with UACR < 30, 21 patients with UACR between 30 and 300, and 8 patients with UACR ≥ 300. We stratified patients into tertiles based on renal FF levels (Tertile 1, Tertile 2, and Tertile 3, *n* = 20 for each group). Demographic, clinical, and biochemical characteristics were compared across the groups. No significant differences were observed among the three groups with respect to age, sex, alcohol consumption, prevalence of hypertension, blood pressure levels, liver and kidney function, or lipid profiles. However, smoking history differed significantly, with the highest proportion of smokers in Tertile 2 (70%) and the lowest in Tertile 3 (30%, *p* = 0.041), suggesting a potential association with renal FF levels. In addition, WC, FPG, and TAT were also significantly different among three groups. Collectively, these findings indicate that individuals with higher renal FF levels are more likely to present with central obesity, impaired glucose metabolism, and pronounced fat accumulation (Table 1).

### 3.2. Correlation Analysis of YKL-40, TNF Receptors, KIM-1 and Fat Distribution

**Figure 1:**
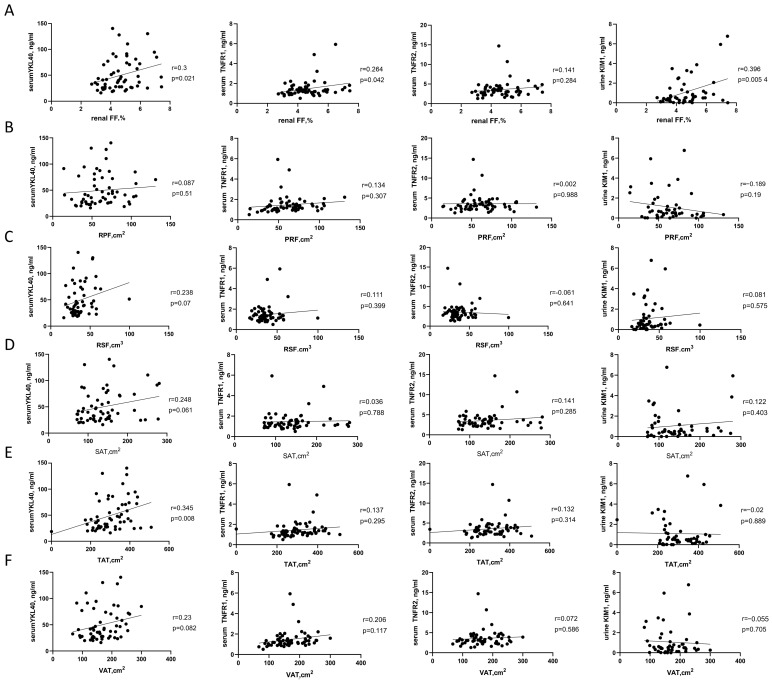
Pearson correlation analyses between adipose tissue depots and inflammatory biomarkers. (**A**) Renal FF; (**B**) PRF; (**C**) RSF; (**D**) SAT; (**E**) VAT; (**F**) TAT. Abbreviations: TNF-R1: Tumor necrosis factor receptors 1, TNF-R2: Tumor necrosis factor receptors 2; YKL-40: Chitinase-3-like protein 1; KIM-1: Kidney injury molecule-1; FF: Fat fraction; TAT: Total adipose tissue; SAT: Subcutaneous adipose tissue; VAT: Visceral adipose tissue; PRF: Perirenal fat.

In this study, serum YKL-40 showed a significant positive correlation with total adipose tissue (TAT) (r = 0.345, *p* = 0.008). Subcutaneous adipose tissue (SAT) and visceral adipose tissue (VAT) also demonstrated positive correlations with YKL-40 (r = 0.248, *p* = 0.061; r = 0.23, *p* = 0.082, respectively), although these did not reach statistical significance. In contrast, perirenal fat (PRF) and renal sinus fat (RSF) showed no significant correlations with YKL-40. Most importantly, serum YKL-40 showed a significant positive correlation with renal FF (r = 0.30, *p* = 0.021). Similarly, TNF-R1 was also positively correlated with renal FF (r = 0.246, *p* = 0.042), while the strongest association was observed between KIM-1 and renal FF (r = 0.396, *p* = 0.004), further supporting the role of lipid accumulation in renal pathological changes. Collectively, these findings indicate that YKL-40, TNF-R1, and KIM-1 are positively associated with renal FF, implying that these biomarkers may play important roles in renal fat accumulation and its related pathological alterations. The relationship between renal fat deposition and inflammatory responses may be mediated, at least in part, through the modulation of these biomarkers, thereby exacerbating renal injury. By contrast, TNF-R2 showed only a weak association, suggesting a relatively limited role in renal fat accumulation and potential regulation by other factors (Figure 1).

### 3.3. Association Between Renal FF and Kidney Injury Biomarkers

Linear regression analysis demonstrated a significant association between renal FF and both inflammatory and kidney injury biomarkers. In the unadjusted model (Model 1), renal FF was positively correlated with serum YKL-40 (β = 0.300, *p* = 0.021), TNF-R1 (β = 0.263, *p* = 0.042), and urinary KIM-1 (β = 0.396, *p* = 0.004), whereas no significant association was observed with TNF-R2 (*p* = 0.284). After adjustment for age, sex, and disease duration in Model 2, the associations of renal FF with YKL-40, TNF-R1, and KIM-1 remained statistically significant. Further adjustment for smoking, alcohol consumption, hypertension, diabetes duration, RAAS inhibitors, SGLT2 inhibitors, GLP-1 receptor agonists, and lipid-lowering drugs in Model 3 showed a statistically significant association between renal FF and YKL-40 (β = 0.275, *p* = 0.025), TNF-R1 (β = 0.327, *p* = 0.015), and urinary KIM-1 (β = 0.352, *p* = 0.021).

After additional adjustment for visceral adipose tissue (VAT) in Model 4, the associations of renal FF with TNF-R1 (β = 0.311, *p* = 0.029) and KIM-1 (β = 0.470, *p* = 0.002) remained significant. We use the Benjamini–Hochberg procedure for false discovery rate (FDR) control in multiple comparisons. The results showed that KIM-1 had significant differences. It indicated that renal fat accumulation is independently associated with systemic inflammation and proximal tubular injury, irrespective of other adipose tissue depots. These findings underscore the potential role of renal FF in the pathogenesis of metabolic abnormalities and renal dysfunction and highlight the need for further investigation into its underlying biological mechanisms and pathological pathways (Table 2).

### 3.4. Lasso Regression Between Renal FF and Kidney Injury Biomarkers

**Figure 2:**
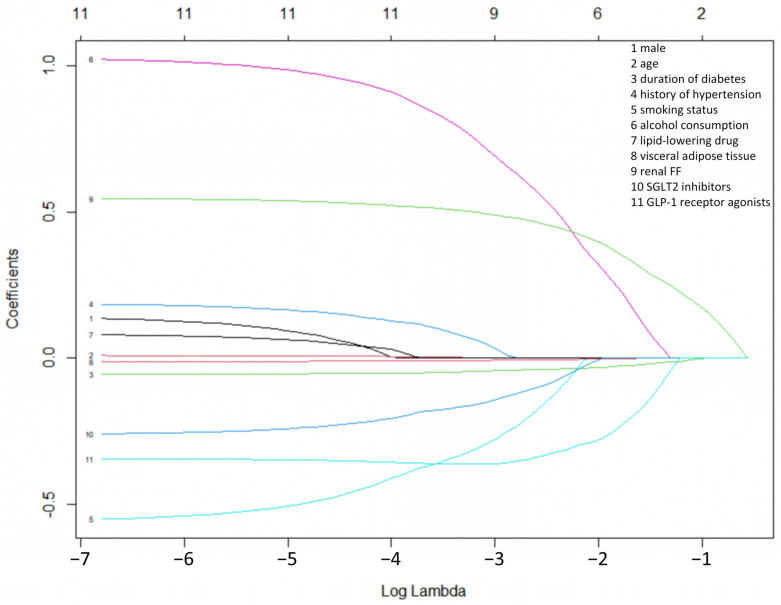

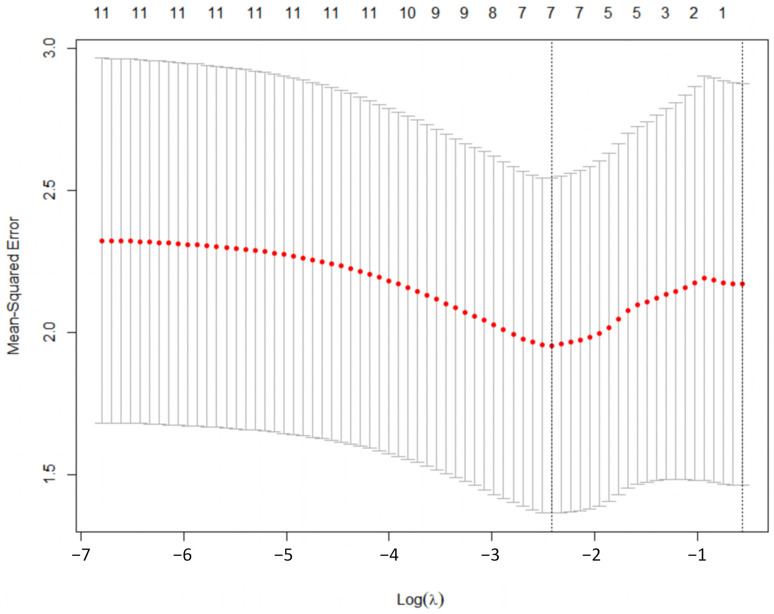
Optimal number of independent variables to model through lasso regression analysis.

As shown in Figure 2, Lasso regression analysis demonstrated the relationship between renal FF and TNF-R1 and KIM-1. Based on the lambda.min threshold, 9 variables with non-zero coefficients were selected in TNF-R1 and KIM-1. Based on the lambda threshold, 9 variables [Renal FF, duration of diabetes, smoking status, Alcohol consumption, RAAS inhibitors, SGLT2 inhibitors, GLP-1 receptor agonists, lipid-lowering drugs, and visceral adipose tissue] with non-zero coefficients were ultimately selected as significant predictors in KIM-1. In Table 3, multivariable linear regression analysis indicated that renal FF (B  =  0.548, *p*  =  0.002), duration of diabetes (B = −0.051, *p*  =  0.085), smoking status (B = −0.479, *p*  =  0.279), alcohol consumption (B = 1.081, *p*  =  0.018), RAAS inhibitors (B = 0.900, *p*  =  0.069), SGLT2 inhibitors (B = −0.376, *p*  =  0.455), GLP-1 receptor agonists (B = −0.693, *p*  =  0.247), lipid-lowering drugs (B = 0.030, *p*  =  0.942), and visceral adipose tissue (B = −0.012, *p*  =  0.008) were independently associated with KIM-1 (adjusted R^2^  =  0.429) (Table 3).

## 4. Discussion

This study aimed to investigate the relationship between renal FF and biomarkers of kidney injury in patients with type 2 diabetes mellitus (T2DM). The results demonstrated significant positive correlations between renal FF and urinary KIM-1, suggesting a close association between renal fat accumulation and kidney injury. In diabetic patients, fat accumulation has been shown not only to be linked with systemic inflammatory responses but also to contribute to local renal injury, thereby exacerbating renal dysfunction [15]. These findings provide novel evidence for the role of lipid deposition in kidney injury and highlight potential biomarkers for early diagnosis.

The lipid nephrotoxicity hypothesis postulates that hyperlipidemia and lipid accumulation induce renal injury through inflammation, excessive reactive oxygen species (ROS) production, and mitochondrial dysfunction [16]. Proximal tubular epithelial cells are the primary sites of lipid deposition, where long-chain saturated fatty acids, oxidized HDL, and BVRA deficiency have all been shown to promote tubular injury and fibrosis via impaired fatty acid oxidation and mitochondrial respiratory dysfunction [17,18]. KIM-1 is a member of the immunoglobulin superfamily. It is known as T-cell immunoglobulin mucin domains (TIM-1). KIM-1 is significantly upregulated in the proximal tubules of damaged or diseased kidneys [19]. A 15-year prospective study showed that blood KIM-1 levels are a good biomarker to predict the rate of eGFR loss and risk of ESRD in patients with type 1 diabetes and proteinuria [20]. Furthermore, Mendelian randomization analysis demonstrated a causal relationship between elevated KIM-1 levels and decreased eGFR. The association is independent of diabetes duration and urinary albumin levels [21].

Compared with existing studies, although some research has examined the impact of fat accumulation on renal function in patients with diabetes [22], quantitative assessment of intrarenal fat and its association with early kidney injury biomarkers remains scarce. The study systematically investigates the relationship between renal FF and early kidney injury biomarkers. These findings not only provide deeper insights into the mechanistic role of renal fat accumulation but also highlight new potential therapeutic targets. Specifically, the significant associations observed between renal fat accumulation and early kidney injury biomarkers suggest that renal FF may serve as an independent biomarker closely linked to renal injury, and this relationship remains robust even after adjusting for traditional metabolic confounders such as smoking, alcohol consumption, and visceral fat. This discovery offers a theoretical basis for fat-targeted intervention strategies, particularly in the treatment of diabetic kidney disease, where fat modulation may represent a novel therapeutic approach. Moreover, by employing MRI to quantitatively evaluate renal FF, this study overcomes the limitations of traditional methods and provides more accurate data on renal fat accumulation.

However, this study has several limitations. First, the study population consisted exclusively of patients with T2DM, and it remains uncertain whether the findings can be generalized to other types of chronic kidney disease. Second, the number of participants is limited; therefore, future studies with larger sample sizes are needed to validate the findings of this study. Third, due to the cross-sectional design, causal relationships between renal FF and long-term changes in renal function could not be established; prospective longitudinal studies are warranted to further validate the prognostic value of renal FF as a marker of renal lipid accumulation in diabetic nephropathy. Although multiple potential confounders were carefully adjusted for, other unrecognized biological mechanisms may still underlie the association between fat accumulation and renal injury. Future investigations should focus on elucidating the causal link between renal FF and renal function decline, as well as evaluating its potential utility as an early diagnostic tool. Moreover, whether fat-targeted interventions can effectively delay the progression of renal injury remains an important question for future research.

## 5. Conclusions

In conclusion, the study demonstrates a significant association between renal FF and early kidney injury biomarkers, highlighting a potentially important role of renal fat accumulation in diabetic nephropathy. These findings provide a theoretical basis for fat-targeted intervention strategies and offer new perspectives for the early diagnosis and clinical management of renal injury.

## Figures and Tables

**Table 1:** Clinical Characteristics of the Study Population Stratified by Tertiles of Renal Fat Fraction (FF).

	All	Tertile 1	Tertile 2	Tertile 3	*p*-Value
	*n* = 60	*n* = 20	*n* = 20	*n* = 20	
renal FF (%)	4.5 (3.6, 5.2)	3.5 (3.2, 3.6)	4.5 (4.2, 4.7)	5.8 (5.2, 6.5)	<0.001
Age (years)	58.0 (51.0, 68.0)	58.0 (49.5, 67.8)	55.0 (49.3, 66.0)	60.0 (53.8, 69.0)	0.445
Duration of diabetes (years)	11.5 ± 6.6	12.7 ± 7.1	10.4 ± 6.7	11.5 ± 6.0	0.546
Male, *n* (%)	45 (75%)	15 (0.8%)	18 (0.9%)	12 (0.6%)	0.091
smoking, *n* (%)	30 (50%)	10 (50%)	14 (70%)	6 (30%)	0.041
Alcohol consumption, *n* (%)	20 (33.3%)	7 (35%)	7 (35%)	6 (30%)	0.928
BMI (kg/m^2^)	25.2 ± 2.8	24.3 ± 2.1	25.0 ± 2.3	26.3 ± 3.4	0.065
WC (cm)	92.6 ± 7.1	90.0 ± 5.2	91.2 ± 6.4	96.5 ± 8.1	0.017
SBP (mmHg)	130.8 ± 17.2	132.9 ± 17.5	125.7 ± 19.0	133.9 ± 14.5	0.261
DBP (mmHg)	79.8 ± 10.4	81 ± 11.8	76.7 ± 10.5	81.7 ± 8.4	0.262
FPG (mmol/L)	7.2 (6.2, 8.8)	7.2 (6.3, 8.8)	6.55 (5.3, 7.3)	8.2 (6.8, 9.9)	0.007
HbA1c (%)	7.4 (6.5, 8.7)	7.3 (6.8, 9.1)	6.8 (5.8, 7.9)	8.1 (6.7, 9.1)	0.073
ALT (U/L)	20.0 (14.3, 26.3)	17.5 (14.5, 23.3)	21.5 (11.5, 26.3)	21.0 (15.5, 31.8)	0.531
ALB (U/L)	45.0 (42.0, 48.0)	45.0 (42.3, 48.8)	45.5 (44.0, 48.0)	43.5 (41.0, 47.0)	0.463
Scr (μmol/L)	88.1 ± 29.5	80.3 ± 24.7	91.9 ± 20.8	92.0 ± 39.5	0.299
BUN (mmol/L)	6.4 (5.2, 7.8)	5.9 (5.2, 7.9)	6.5 (5.1, 7.3)	6.5 (5.2, 8.5)	0.802
eGFR (CKD-EPI)	81.3 ± 22.1	88.0 ± 21.8	79.2 ± 20.2	76.8 ± 23.6	0.242
UACR (mg/g)	24.0 (10.4, 80.8)	33.7 (13.2, 71.9)	16.6 (8.8, 55.9)	49.4 (10.9, 147.1)	0.260
Uric acid (mmol/L)	347.0 ± 105.3	315.4 ± 80.1	389.2 ± 116.0	336.6 ± 107.4	0.072
TG (mmol/L)	1.6 (1.1, 2.3)	1.5 (1.0, 2.2)	1.2 (0.8, 2.3)	1.6 (1.2, 2.6)	0.425
TC (mmol/L)	3.9 (3.2, 5.0)	4.0 (3.3, 5.5)	4.0 (3.5, 4.6)	3.8 (3.0, 5.1)	0.715
HDL (mmol/L)	1.1 ± 0.3	1.1 ± 0.3	1.1 ± 0.4	1.1 ± 0.3	0.710
LDL (mmol/L)	2.2 (1.7, 3.0)	2.3 (1.8, 3.7)	2.2 (1.7, 2.8)	2.0 (1.5, 3.1)	0.720
INS (%)	26 (43.3%)	8 (40%)	8 (40%)	10 (50%)	0.762
ACEI/ARB (%)	20 (33.3%)	6 (30%)	5 (25%)	9 (45%)	0.377
anti-lipid (%)	29 (48.3%)	9 (45%)	10 (50%)	10 (50%)	0.935
SGLT-2i	12 (20%)	3(15%)	3(15%)	6(30%)	0.428
GLP-1RA	21 (35%)	9(45%)	7(35%)	5(25%)	0.471
TAT (cm^2^)	303.5 ± 86.8	278.1 ± 62.7	280.8 ± 98.9	351.7 ± 77.5	0.008
SAT (cm^2^)	135.5 (99.7, 159.1)	114.8 (99.1, 151.9)	135.5 (88.0, 154.0)	147.8 (119.5, 238.2)	0.050
VAT (cm^2^)	168.0 ± 51.5	154.3 ± 47.9	167.8 ± 49.6	182.0 ± 55.5	0.241
PRF (mm)	61.7 ± 23.8	58.0 ± 26.1	59.3 ± 25.2	67.7 ± 19.7	0.388

Abbreviations: BMI: Body mass index; WC: Waist Circumference; SBP: Systolic blood pressure; DBP: Diastolic blood pressure; FPG: Fasting plasma glucose; HbA1c: Glycated hemoglobin A1c; ALT: Alanine Aminotransferase; ALB: Albumin; Scr: Serum creatinine; BUN: Blood Urea Nitrogen; eGFR: Estimated glomerular filtration rate; UACR: Urinary albumin-to-creatinine ratio; TG: Triglyceride; TC: Total cholesterol; HDL: High density lipoprotein; LDL: Low density lipoprotein; INS: Insulin; ACEI: Angiotensin converting enzyme inhibitors; TAT: Total adipose tissue; SAT: Subcutaneous adipose tissue; VAT: Visceral adipose tissue; PRF: Perirenal fat.

**Table 2:** Associations Between Renal Fat Fraction (Renal FF) and Kidney Injury Biomarkers Based on Linear Regression Models.

Model	Model 1		Model 2		Model 3		Model 4	
	β	*p* Value	β	*p* Value	β	*p* Value	β	*p* Value
serum YKL-40	0.300	0.021	0.279	0.028	0.275	0.025	0.218	0.083
serum TNF-R1	0.263	0.042	0.263	0.044	0.327	0.015	0.311	0.029
serum TNF-R2	0.141	0.284	0.136	0.303	0.203	0.164	0.209	0.178
urine KIM-1	0.396	0.004	0.405	0.004	0.352	0.021	0.470	0.002

Model 1: unadjusted. Model 2: adjusted for age, sex, and duration of diabetes. Model 3: further adjusted for smoking status, alcohol consumption, history of hypertension, RAAS inhibitors, SGLT2 inhibitors, GLP-1 receptor agonists, and lipid-lowering drugs. Model 4: Additionally adjusted for visceral adipose tissue. Abbreviations: TNF-R1: Tumor necrosis factor receptors 1, TNF-R2: Tumor necrosis factor receptors 2; YKL-40: Chitinase-3-like protein 1; KIM-1: Kidney injury molecule-1; RAAS: Renin-angiotensin-aldosterone system; SGLT2: Sodium-dependent glucose transporters 2; GLP-1: Glucagon-Like Peptide-1.

**Table 3:** Multivariable associations between Renal Fat Fraction (Renal FF) and KIM-1.

	KIM-1	
	B	*p* Value
Renal FF	0.548	0.002
Duration of diabetes	−0.051	0.085
Smoking status	−0.479	0.279
Alcohol consumption	1.081	0.018
RAAS inhibitors	0.900	0.069
SGLT2 inhibitors	−0.376	0.455
GLP-1 receptor agonists	−0.693	0.247
Lipid-lowering drugs	0.030	0.942
Visceral adipose tissue	−0.012	0.008

Abbreviations: KIM-1: Kidney injury molecule-1; RAAS: Renin-angiotensin-aldosterone system; SGLT2: Sodium-dependent glucose transporters 2; GLP-1: Glucagon-Like Peptide-1.

## Data Availability

The data that support the findings of this study are available from the corresponding author upon reasonable request.
